# Identification of ellagic acid and urolithins as natural inhibitors of Aβ_25–35_-induced neurotoxicity and the mechanism predication using network pharmacology analysis and molecular docking

**DOI:** 10.3389/fnut.2022.966276

**Published:** 2022-08-02

**Authors:** Hui-Lin Li, Shi-Ying Zhang, Ying-Shan Ren, Jie-Chun Zhou, Ying-Xin Zhou, Wei-Zhong Huang, Xiu-Hong Piao, Zhi-You Yang, Shu-Mei Wang, Yue-Wei Ge

**Affiliations:** ^1^School of Chinese Materia Medica, Guangdong Pharmaceutical University, Guangzhou, China; ^2^Key Laboratory of Digital Quality Evaluation of Chinese Materia Medica of State Administration of TCM, Guangdong Pharmaceutical University, Guangzhou, China; ^3^Engineering and Technology Research Center for Chinese Materia Medica Quality of the Universities of Guangdong Province, Guangdong Pharmaceutical University, Guangzhou, China; ^4^Macau University of Science and Technology, Macau, Macau SAR, China; ^5^Guangdong Luofushan Sinopharm Co., Ltd., Huizhou, China; ^6^School of Life Sciences and Biopharmaceutics, Guangdong Pharmaceutical University, Guangzhou, China; ^7^Guangdong Provincial Key Laboratory of Aquatic Product Processing and Safety, College of Food Science and Technology, Guangdong Ocean University, Zhanjiang, China

**Keywords:** Alzheimer’s disease, ellagic acid, urolithins, neuroprotection, synaptic plasticity, natural inhibitor

## Abstract

Ellagic acid (EA) is a dietary polyphenol that widely exists in grapes, strawberries, and walnuts. It usually exerts multiple biological activities together with its *in vivo* metabolites called urolithins. EA and urolithins had been proposed as natural agents for applying on the early intervention of Alzheimer’s disease (AD). However, the neuroprotective effects of those small molecules have not been confirmed, and the action mechanism is not clear. Deposition of beta-amyloid (Aβ) protein is well documented as being involved in the initiation and pathological process of AD. In the present study, we investigated the attenuating effects of EA and several urolithins on Aβ_25–35_-induced neuronal injury and its underlying molecular mechanism by constructing the *in vitro* AD cell model of PC12 cells and primary neurons. The results revealed that EA and urolithins especially the UM5 and UM6 exerted promising neuroprotective effects in improving the Aβ_25–35_-induced cell damage and lactate dehydrogenase (LDH) leakage, reducing reactive oxygen species (ROS) production, inhibiting neuronal apoptosis, and promoting neurite outgrowth. These results provide new insights into the development of UM5 and UM6 as anti-AD candidates. A network pharmacology analysis combining molecular docking strategy was further adopted to predict the signaling pathway involved in the anti-AD action of EA and urolithins, and the activation of PI3K-Akt, as well as the inhibition of MAPK was found to be involved.

## Introduction

Alzheimer’s disease (AD) has become a worldwide disease that endangers human health and incurs heavy economic burden to the health care system. One of its main pathological features is aggregated plaques consisting of Aβ peptides, which have been proven to be toxic to neurons, causing neuronal apoptosis, synaptic loss, and cytoskeleton destruction (1, 2). Therefore, inhibition of Aβ-induced neurotoxicity is one of the classic strategies for the treatment of AD (3). Although we have a better understanding of the pathophysiological process of AD in the past decades, there are only a few drugs can delay the progress of the disease. The treatment of AD is limited and palliative, and the need for clinically effective treatment remains unmet (4). Therefore, research and development of new treatments are urgently needed. In recent years, the natural constituents from food assisting in AD treatment have been expected since their low toxicity and multiple bioactivities (5).

Increasing attention has been paid to natural products and their bioactive metabolites due to a growing appreciation of their role in drug discovery for anti-aging and aging-related diseases (6). Ellagic acid (EA), a dietary polyphenol, distributed widely in the grapes, strawberries, raspberries, cranberries, pomegranates, walnuts, pecans and green tea, and has been focused as anti-AD molecule for its promising neuroprotective activities (7–9). However, it exhibits limited bioavailability *in vivo* because of its flat structure-induced low solubility (10). Usually, EA can be hydrolyzed and metabolized by gut microbiota to generate dibenzopyran-6-one derivatives, called as urolithins such as urolithin M5 (UM5), urolithin M6 (UM6), urolithin A (UA), and urolithin B (UB) that are easily absorbed into blood circulation (11). EA has been recognized posing potential neuroprotective effects by its anti-inflammation, anti-oxidation, and anti-neurotoxicity properties (12). However, whether its major metabolites urolithins have comparable neural beneficial effect has not been clarified, especially for the UM5 and UM6 that have been rarely studied so far.

**FIGURE 1:**
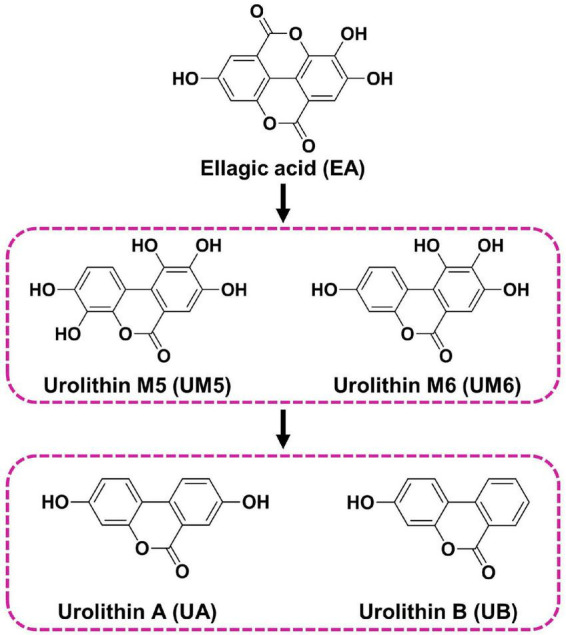
Chemical structures of EA and urolithins evaluated in the study.

In the present study, the protective activity of EA and its four urolithin metabolites (Figure 1) on Aβ_25–35_-induced neurotoxicity was investigated, and the possible mechanisms, especially the synergistic mechanism between various urolithins and EA involved in their anti-AD action were discussed on the basis of network pharmacology. This study will provide a scientific basis for the application of EA and urolithins to the intervention of neurodegenerative disease.

## Materials and methods

### Materials

RPMI Medium 1640 basic(1X), fetal bovine serum (FBS), horse serum (HS), neurobasal™ medium, B-27™ supplement (50 × ), glucose solution, L-glutamine (200 mM) were purchased from Gibco^®^ (Thermo Fisher Scientific, NY, United States). Recombinant Human β-Nerve Growth Factor (NGF, 98% pure) was obtained from Peprotech (Cranbury, NJ, United States). Amyloid β-protein fragment 25–35 (Aβ_25–35_, 97% pure) and 3-(4,5-dimethyl-2-thiazolyl)-2,5-diphenyl-2-H-tetrazolium bromide (MTT), Triton X-100, paraformaldehyde, goat serum and 4-6-diamidino-2-phenylindole (DAPI) were from Beijing Solarbio Science and Technology Co., Ltd. (Beijing, China). A lactate dehydrogenase (LDH) assay kit was purchased from Nanjing Jiancheng Bioengineering Institute (Nanjing, China). The reactive oxygen species (ROS) assay kit and mitochondrial membrane potential (MMP) assay kit with JC-1 were obtained from the Beyotime Institute of Biotechnology (Nanjing, China). The anti-MAP2 (phospho S136) antibody, goat anti-rabbit IgG H&L (Alexa Fluor^®^ 488) were obtained from Abcam (Cambridge, United Kingdom).

### Cell culture and treatment

PC12 cells were purchased from the China Center for Type Culture Collection (Wuhan, China) and cultured in 1,640 medium supplemented with 10% (v/v) HS and 5% (v/v) FBS at 37°C in a saturated humidity incubator containing 5% CO_2_. UA, UB, UM5, UM6, and EA were obtained from Sigma Chemical Co. (St. Louis, United States).

In this study, PC12 cells were seeded in poly-D-lysine (PDL) precoated plates and treated with 100 ng/mL NGF for 5 days for differentiation. Cells were divided into the following groups: (a) control group (Cont), (b) Aβ_25–35_ (5 μM Aβ_25–35_ for 24 h), (c) UA (0.1 and 1 μM with 5 μM Aβ_25–35_), (d) UB (0.1 and 1 μM with 5 μM Aβ_25–35_), (e) UM5 (0.1 and 1 μM with 5 μM Aβ_25–35_), (e) UM6 (0.1 and 1 μM with 5 μM Aβ_25–35_), and (f) EA (0.1 and 1 μM with 5 μM Aβ_25–35_). Except the control group, each group was pre-stimulated with 5 μM Aβ_25–35_ for 24 h, and compounds were treated for another 24 h. While the control group was cultured in normal differentiation medium of 100 ng/mL NGF without Aβ_25–35_ or compounds.

### Determination of mitochondrial membrane potential

The measurement of MMP was performed by using the MMP assay kit with JC-1 according to the manufacturer’s instructions. Cells were incubated with JC-1 staining solution for 20 min at 37°C and the MMP staining was observed under an inverted fluorescence microscope (Olympus, Japan). The fluorescence intensities of JC-1 monomer (Ex/Em = 490/530) and JC-1 polymer (Ex/Em = 525/590 nm) were measured by SPARK multimode microplate reader (Tecan, Trading AG, Switzerland). The red/green fluorescence ratio represents the MMP of PC12 cells.

### MTT assay

Cell viability was determined by using a conventional MTT assay. Briefly, PC12 cells were seeded in pre-coated 96-well plates at a density of 1 × 10^4^ cells per 100 μL, and the medium was changed every 2 days. The cells were treated with drugs in the presence or absence of Aβ for 24 or 48 h. Aβ_25–35_ was dissolved in sterile water and preincubated for 7 days at 37°C for aggregation. Subsequently, the MTT solution (20 μL, 5 mg/mL) was added and incubated at 37°C for 4 h. After carefully removing the medium, 150 μL of DMSO was added to each well to dissolve formazan for 15 min. The optical density (OD) was measured at an absorbance wavelength of 570 nm using a plate reader (Bio-Rad Laboratories Inc., Hercules, CA, United States). The absorbance of control cells was assumed to be 100%, and the survival rate of PC12 cells was calculated accordingly.

### Measurement of intracellular reactive oxygen species

Briefly, PC12 cells were seeded into 6-well plates at a density of 2 × 10^5^ cells and treated as described above. The intracellular ROS level was detected by fluorescent dye 2,7-dichlorofluorescein diacetate (DCFH-DA). In the presence of ROS, DCFH-DA was oxidized to fluorescent dichlorofluorescein (DCF). Ten micromolar DCFH-DA was added to the cells, followed by incubation for 30 min at 37°C in the dark. By using a SPARK multimode microplate reader (Tecan, Trading AG, Switzerland), the fluorescence intensity of DCF was measured at excitation and emission wavelengths of 488 and 525 nm, respectively. The expression of intracellular ROS level was normalized to the control level.

### Lactate dehydrogenase leakage rate analysis

For the LDH assay, PC12 cells were seeded into 6-well plates with a cell density of 2.0833 × 10^4^ cells/cm^2^, and the cells were treated as described above. Then, the culture supernatant was collected and analyzed using a commercial LDH kit, according to the manufacturer’s instructions. The absorbance was detected at 450 nm. The LDH leakage rate was normalized to the control.

### Immunofluorescence detection

PC12 cells were plated into 24-well plates at a density of 2,000 cells/well. After 7 days of differentiation as described above, the immunofluorescence was assessed to evaluate the neurite density. First, cells were fixed with 4% paraformaldehyde at room temperature for 60 min and washed with 0.2% Triton X-100 three times. Subsequently, PC12 cells were immunostained at 4°C overnight with the anti-MAP2 antibody (dilution 1:1,000) containing 1% goat serum and 0.5% Triton X-100, and the cells were washed again with 0.2% Triton X-100, followed by incubation with goat anti-rabbit IgG H&L (dilution 1:500, secondary antibody). Cell nuclei were stained with 4′,6-diamidino-2-phenylindole (DAPI, 1 μg/mL). The fluorescent images were visualized using a fluorescence microscope system (BX61/DP70, Olympus). ImageJ software (NIH, Bethesda, MD, United States) was used for quantitative analysis.

### Primary cortical culture

The embryos were isolated from pregnant ICR mice (China, GDMLAC, Guangzhou, China) at 14 days of gestation. The cortices were dissected, and the dura mater was removed. The tissue was minced into 1 × 1 × 1 mm^3^ pieces, followed by separation and culture in the neurobasal medium supplemented with 1 × B-27 supplement (50 × ), 0.6% D-glucose, and 2 mM L-glutamine in 24-well plates precoated with PDL at 37°C in a saturated humidity incubator containing 5% CO_2_.

### Network pharmacology analysis

The chemical structures of EA and urolithins were downloaded by PubChem.^1^ Subsequently, the 3D structures were imported into SwissTargetPrediction^2^ and PharmMapper^3^ to match the targets. AD-associated targets were collected from GeneCards^4^ and the OMIM database.^5^ The intersection targets were obtained by using Venny 2.1.0 and were then sent for analysis online using STRING 11.5 to construct the protein-protein interaction (PPI) network. Gene Ontology (GO) and Kyoto Encyclopedia of Genes and Genomes (KEGG) pathway enrichment analysis were completed by Metascape.^6^ Finally, the “component-target-pathway” (C-T-P) network was constructed via Cytoscape 3.9.0 software. The associations among components, candidate targets, and AD-related pathways were analyzed by the cytoHubba plug-in in Cytoscape 3.9.0 software. The corresponding nodes were ranked according to the MCC algorithm to screen the hub genes.

### Molecular docking studies

The molecular docking analysis was performed via SYBYL-X 2.1. The chemical structures of EA and urolithins were downloaded from PubChem (see text footnote 1). The crystal structures of key targets were obtained from the Protein Data Bank.^7^ The protein and ligand interactions were analyzed after removing water molecules, and hydrogen atoms were added. The results of molecular docking are shown in Table 1. The binding sites and binding patterns between the molecule and the target were automatically generated and visualized using Pymol-2.5.2.

**TABLE 1:** The docking results of ellagic acid and urolithins with key targets.

Drug	Gene name	PDB ID	Score
UA	AKT1	6ccy	6.168
UB	AKT1	6ccy	6.367
UM5	AKT1	6ccy	4.943
UM6	AKT1	6ccy	3.744
EA	AKT1	6ccy	6.170
UA	IGF1R	2oj9	6.844
UB	IGF1R	2oj9	6.285
UM5	IGF1R	2oj9	8.594
UM6	IGF1R	2oj9	5.678
EA	IGF1R	2oj9	5.837
UA	NFKB1	1ikn	5.241
UB	NFKB1	1ikn	5.084
UM5	NFKB1	1ikn	3.618
UM6	NFKB1	1ikn	4.203
EA	NFKB1	1ikn	4.407
UA	EGFR	4i24	5.525
UB	EGFR	4i24	5.690
UM5	EGFR	4i24	4.685
UM6	EGFR	4i24	3.891
EA	EGFR	4i24	5.929
UA	MAPK14	5wjj	6.726
UB	MAPK14	5wjj	6.255
UM5	MAPK14	5wjj	8.258
UM6	MAPK14	5wjj	7.740
EA	MAPK14	5wjj	7.547

### Statistical analysis

All experiments were performed in triplicate. GraphPad Prism version 9 (GraphPad Software, San Diego, California, United States) was adopted to perform a one-way analysis of variance (ANOVA) with the *post hoc* Dunnett’s test. Values are expressed as means ± standard deviations (SDs), with a value of *p* < 0.05 indicating statistic significant significance.

## Results and discussion

### Ellagic acid and urolithins reverse Aβ_25–35_-induced MMP depolarization

Mitochondria are the center of cell energy production, mainly responsible for ATP production, ROS formation, intracellular calcium homeostasis, and apoptosis (13). In AD, increased ROS and loss of MMP have been observed (14). The decline of MMP is considered to be a hallmark event of the early apoptotic cascade in cells, and the collapse of mitochondrial transmembrane potential (ΔΨ*m)* also indicates that apoptosis is irreversible (15). Normally, the JC-1 probe aggregates in the mitochondrial matrix and emits red fluorescence, while JC-1 acts as a green fluorescent monomer in the cytoplasm when cells are damaged.

**FIGURE 2:**
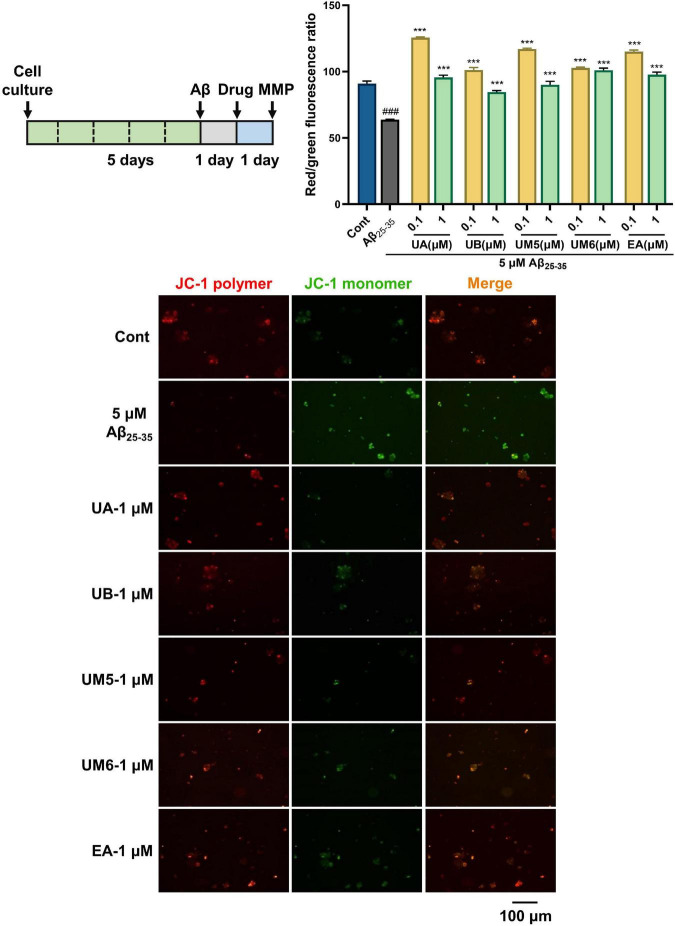
EA and urolithins restored mitochondrial transmembrane potential and inhibited apoptosis. PC12 cells were incubated with JC-1 probe and observed and photographed under a fluorescence microscope. Red indicates the fluorescence emitted by the JC-1 polymer, and green indicates the fluorescence emitted by the JC-1 monomer. The red/green fluorescence ratio represents the MMP of PC12 cells. Data presented as mean ± S.D., *n* = 3, ###*p* < 0.001 vs. Cont; ***p < 0.001 vs. Aβ_25–35_; Scale bar = 100 μm.

As shown in Figure 2, in the present study, the control group exhibited strong red fluorescence, indicating the high cell membrane potential. After being exposed to Aβ_25–35_ for 24 h, the red/green fluorescence ratio of PC12 cells decreased significantly, revealing that Aβ_25–35_ promoted the decrease in MMP and impaired mitochondrial function (*p* < 0.001). In contrast, MMP levels were significantly elevated or even reversed after treatment with EA and urolithins (*p* < 0.001). In particular, we found that low concentrations of EA and urolithins positively increased ΔΨ*m* and reversed Aβ-induced mitochondrial depolarization. The reason for this may be that low-concentration compound stimulation promotes the energy conversion of cells. Some similar results can be found, where the blocking activity of 41 small molecules on Aβ-induced mitochondrial permeability transition pore (mPTP) opening, i.e., the ability to restore MMP, were evaluated by JC-1 assay measuring changes in ΔΨ*m*. Among them, 25 compounds exhibited better inhibitory activity in inhibiting Aβ-induced mPTP opening than cyclosporin A (CsA) as a standard control (16). Overall, the compounds exhibited a certain protective effect on mitochondrial damage caused by Aβ.

Mitochondrial dysfunction is involved in the pathogenesis of neurodegenerative diseases such as AD and is characterized by alterations in mitochondrial bioenergetics, including the loss of MMP (17). However, the effects of urolithins vary depending on the model used in the study. Ryu et al. found that although the lifespan of *C. elegans* treated with 50 μM UA was significantly prolonged while the MMP level was decreased. The author explained that the loss of MMP may be related to the enhancement of mitochondrial mitophagy (18). While Esselun et al. observed that 1 μM UA had no significant effect on MMP level in control cell lines (SH-SY5Ymock) and early AD models (SH-SY5Y-APP695). Conversely, a 10-fold increased level of UA (10 μM) yielded no observable improvement other than model-specific changes and even deteriorated mitochondrial function (19). In this study, we constructed the AD model of PC12 cells *in vitro*, and the difference in the regulation of MMP caused by different models cannot be ignored. Therefore, other mitochondrial parameters should also be considered and included in the scope of investigation, such as increased oxidative stress markers and Aβ production and decreased ATP level and oxygen consumption (20). Through these additional investigations, further systematic and in-depth research can be carried out, which we aim to perform as our follow-up work.

### Ellagic acid and urolithins ameliorate Aβ_25–35_-induced cytotoxicity

**FIGURE 3:**
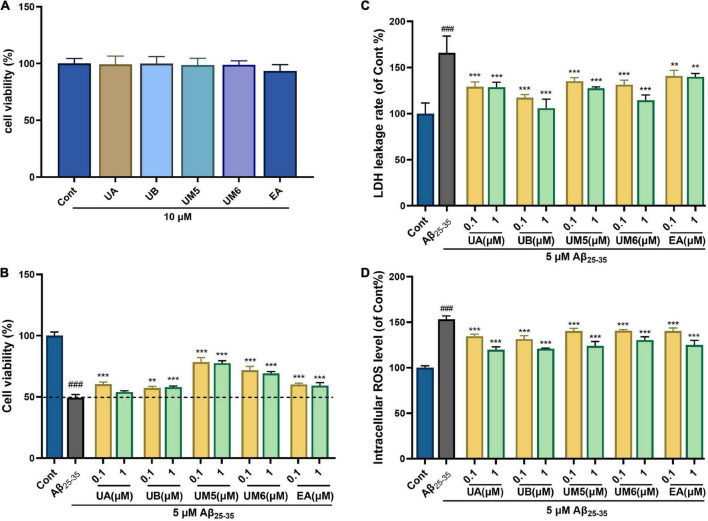
Effects of EA and urolithins against Aβ_25–35_-induced injury. **(A)** Cytotoxicity of EA and urolithins in PC12; **(B)** protective effect of EA and urolithins in Aβ_25–35_-injured PC12 cells; **(C)** inhibition of Aβ_25–35_-induced lactate dehydrogenase (LDH) leakage rate by EA and urolithins; **(D)** inhibitory effect of EA and its metabolites on intracellular ROS level induced by Aβ_25–35_. Data presented as mean ± S.D., *n* = 6, ###*p* < 0.001 vs. Cont; **p < 0.01 vs. Aβ_25–35_; ***p, 0.001.

To investigate the effects of EA and urolithins on normal cell proliferation, toxicity tests were performed at a higher extreme concentration than in this study. As shown in Figure 3A, the tested compounds did not exhibit any apparent cytotoxicity at a 10 μM concentration.

#### Protective effect of ellagic acid and urolithins against Aβ_25–35_-induced cell death

Our data showed that the cell viability of the model group was significantly decreased after being treated with 5 μM Aβ_25–35_ for 24 h (Figure 3B, *p* < 0.001). Aβ_25–35_-induced cell death was significantly inhibited following treatment with compounds for 24 h. The UB group exhibited a dose-dependent manner. Strikingly, the UM5 and UM6 groups showed significantly increased survival rates of PC12 cells compared with the other groups (*p* < 0.001). Overall, the cell viability was significantly improved by the treatment compounds in the AD cell model, indicating the cytoprotective activities of EA and urolithins. Notably, our data revealed that UM5 and UM6 could play a better protective role and inhibit cell death induced by Aβ_25–35_ compared with UA and UB, which might be related to the position and number of hydroxyl groups in the side chain. It provides a new perspective for the study of AD intervention drugs.

#### Ellagic acid and urolithins attenuate the release of cellular lactate dehydrogenase

The degree of nerve cell injury is proportional to the release of LDH. To assess the effect of EA and urolithins on Aβ_25–35_-induced neurotoxicity, the LDH enzyme activity was determined. Compared with the control group, the LDH release in the model group was significantly increased (Figure 3C, *p* < 0.001), indicating that the toxicity produced by Aβ_25–35_ altered the permeability of the cell membrane and, therefore, resulted in a significant release of LDH. Meanwhile, we found that the LDH enzyme activity in compound- treated groups was lower than that in the model group, but not in a dose-dependent manner. The results demonstrated an average and significant inhibiting effect of EA and urolithins in LDH leakage compared with the model group (Figure 3C, *p* < 0.01).

The release of LDH is correlated with the degree of cell damage (21). Taken together, the results indicated that UB had a good protective effect on the cytotoxicity against Aβ_25–35_, which may be achieved by protecting the selective permeability of the cell membrane and maintaining the homeostasis of the intracellular environment. Similarly, the results of the MTT and LDH experiments reported by Ahsan et al. showed that UA alleviated OGD/R-induced injury in N2a cells and primary neurons but not in a concentration dependent manner (22). In summary, MTT and LDH assays were conducted to comprehensively evaluate the cytotoxicity and activity of compounds, and the results confirmed that the compounds could exert neuroprotective effects without affecting or damaging the cells.

#### Ellagic acid and urolithins inhibit Aβ_25–35_-induced intracellular reactive oxygen species accumulation

To evaluate the effects of EA and urolithins on Aβ_25–35_-induced oxidative stress, ROS levels were measured. As shown in Figure 3D, intracellular ROS levels were significantly increased after 24 h of stimulation with 5 μM Aβ_25–35_, which suggests that Aβ_25–35_ induces oxidative stress. After treatment with different concentrations of compounds (0.1 and 1 μM), intracellular ROS levels were significantly inhibited, and the normal oxidation-reduction reaction (REDOX) state of cells was improved and restored to a certain extent.

Lee’s group revealed that UB could exert an antioxidant effect by reducing ROS production and NADPH oxidase subunit expression (23), which is consistent with our data. Moreover, our findings also suggested that, in addition to UB, EA, UA, UM5, and UM6 could all maintain the balance of intracellular oxidation and antioxidant systems to a certain extent, and mitigate ROS-mediated oxidative stress. The antioxidant effect of each compound was uniform and the trend was approximately the same. Accordingly, we speculated that both EA and urolithins can inhibit mitochondrial damage and early apoptosis by restoring ΔΨ*m* levels, improving Aβ_25–35_-induced cytotoxicity, and reducing intracellular ROS accumulation, thereby achieving neuroprotective effects.

### Ellagic acid and urolithins ameliorate Aβ_25–35_-induced neurite atrophy

**FIGURE 4:**
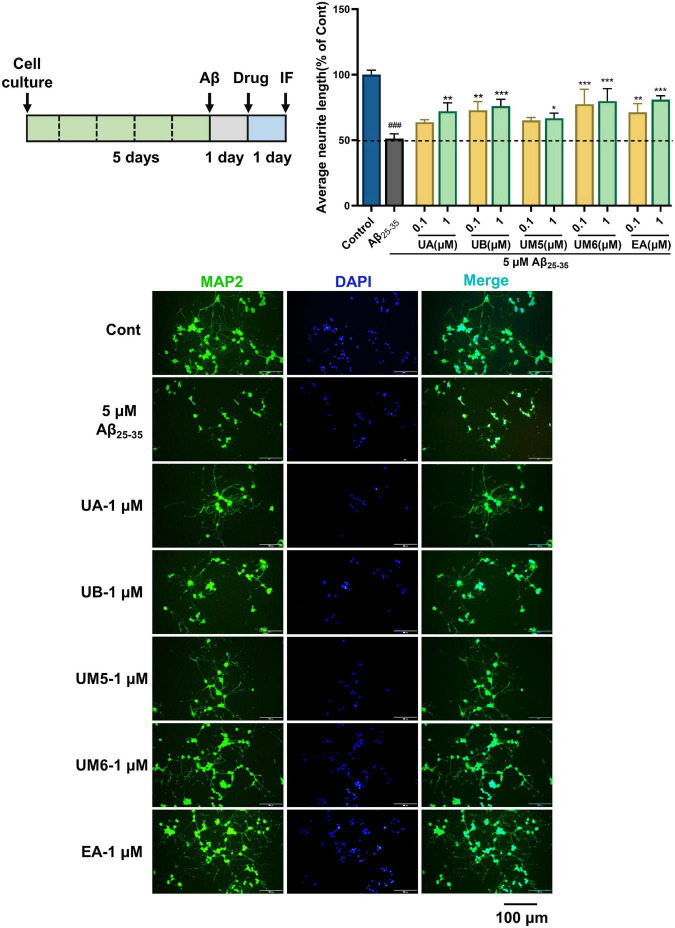
Effects on Aβ_25–35_-induced neurite atrophy in PC12. The PC12 cells were differentiated for 5 days, and then the cells were treated with (Aβ_25–35_) or without (Cont) 5 μM Aβ_25–35_ for 24 h. Each compound at a concentration of 0.1 or 1 μM containing NGF (100 ng/mL), or Aβ_25–35_ was added to the neurons and cultured for another 24 h after removing the Aβ-containing medium. Then the cells were fixed and immunostained with MAP2 and DAPI. The lengths of the MAP2 positive neurites were measured. The values are the means ± S.D. of the data. *n* = 3. ### *p* < 0.001 vs. Control, *p < 0.05 vs. Aβ_25–35_, **p < 0.01, ***p < 0.001. Scale bar = 100 μm.

Synaptic plasticity is considered to be an important neurochemical basis for memory and learning (24). Therefore, the regenerative activity of neurites is of great significance in AD therapy. To investigate the protective effects of EA and urolithins on neurons, we constructed a PC12 cell injury model induced by Aβ_25–35_. MAP2 was used to specifically label the neurites of neurons, and the effects of each compound on Aβ_25–35_-induced neurite atrophy were observed and analyzed. When exposed to Aβ, the neuronal processes shrank significantly, the number of adherent cells decreased, synaptic connectivity between cells decreased, and neuronal networks were disrupted (Figure 4). Our data indicated that the average neurite length of PC12 cells was significantly decreased after 24 h of treatment with Aβ_25–35_ (*p* < 0.001). After another 24 h of treatment with the compounds, all groups exhibited an inhibition of neurite atrophy in a dose-dependent manner. Notably, the high-concentration group (1 μM) exhibited the preferable therapeutic effect (*p* < 0.05). Currently, there are no supporting data or research report related to the regulation of synapse outgrowth by EA and urolithins. Therefore, we performed immunofluorescence staining using the neuronal marker MAP2 to observe the morphological changes of neurons with neurite atrophy caused by Aβ-induced toxicity, and the effects of these compounds on synaptic growth were investigated for the first time. From this analysis, we reasoned that this neuroprotective activity may be achieved by promoting the regrowth of atrophic synapses. However, further verification is still required.

### Ellagic acid and urolithins promote neurite regeneration against Aβ_25–35_-induced neurite atrophy in primary cortical neurons

**FIGURE 5:**
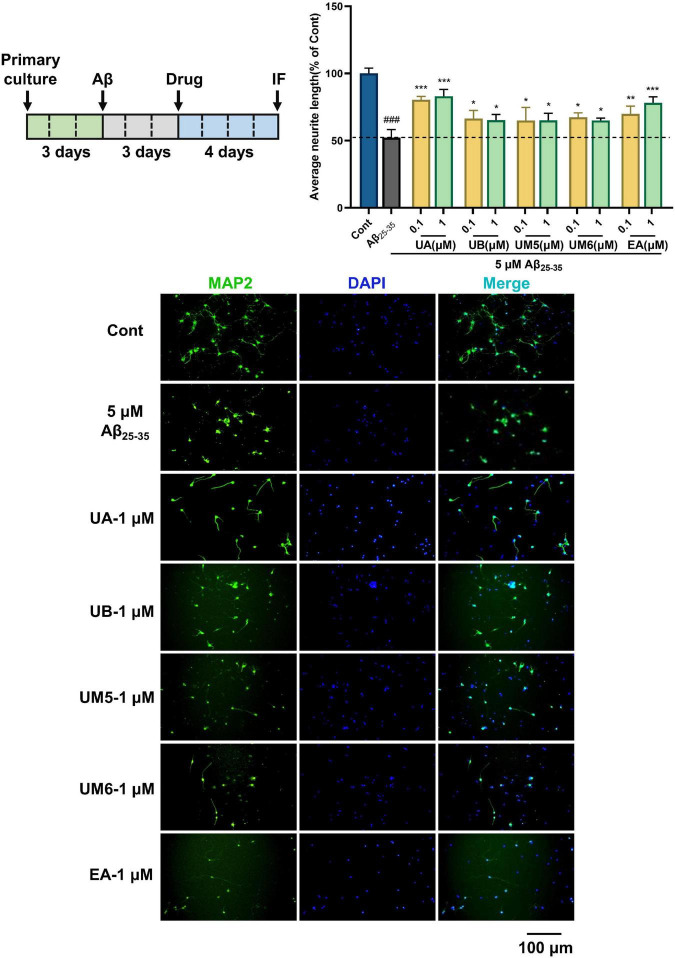
Effects on Aβ_25–35_-induced neurite atrophy in primary cortical neurons. Cortical neurons were cultured for 3 days followed treatment with Aβ_25–35_ for another 3 days, and then treated with drugs (0, 0.1, or 1 μM) 4 days after removing the Aβ-containing medium. The cells were double immunostained with MAP2 and DAPI. The MAP2-possitive neurons were quantified. *n* = 3. ###*p* < 0.001 vs. Control, *p < 0.05 vs. Aβ_25–35_, **p < 0.01, ***p < 0.001. Scale bar = 100 μm.

To further confirm the neuroprotective effect in the PC12 cells, we established the Aβ_25–35_-induced neurite atrophy model in primary cortical neurons, and assessed the neurite regenerative effects of EA and urolithins. As illustrated in Figure 5, compared with the control group (Cont), the average neurite length of the model group (Aβ_25–35_) was significantly decreased (*p* < 0.001), demonstrating that Aβ_25–35_ significantly inhibited the outgrowth of neuronal neurites. After 4 days of drug treatment, the average neurite lengths of the UA, UB, UM5, UM6, and EA groups were significantly increased (*p* < 0.05), suggesting that EA and urolithins significantly promoted the outgrowth of MAP2-positive neurites. In particular, the UA and EA groups exhibited a dose-dependent therapeutic effect (*p* < 0.01). These results further verified the effects of EA and urolithins on neuronal neurite regeneration and revealed that they can improve the synaptic plasticity of neurons against Aβ-induced cell damage.

In immunofluorescence experiments, we found that compound UM6 exhibited a better neuroprotective effect on PC12 cells, whereas the UA exhibited better activity in primary cortical neurons. Although the neurite regrowth effect of compounds did not show a significant dose-dependent trend, it is undeniable that the neural network reconstruction was observed in both cell models. Nevertheless, the activity trends of compounds in the two models were not consistent. Contrary to the concentration-dependent experimental trend at the PC12 cell level, results at the primary cortical neurons level showed uniform neuroprotective activity. We speculate that this may likely be due to the difference in the sensitivity of cell lines and primary neurons, leading to differences in drug treatment efficacy and sensitivity. Overall, both EA and urolithins exert positive effects on neurite protection and neurite regeneration.

### Predication of action mechanism through network pharmacology

Based on the existing database, network pharmacology integrates network biology and polypharmacology, which provides a novel research approach for exploring the mechanism and synergistic effect of compounds on disease treatment. To further reveal the potential targets and related molecular mechanisms of EA and its urolithin metabolites in AD intervention, we utilized network pharmacology to conduct a systematic analysis. The SwissTargetPrediction, PharmMapper, GeneCards, and OMIM databases were searched, revealing 343 drug targets and 1,856 disease targets. Moreover, a total of 131 AD-related drug targets were mapped by importing the data into Venny 2.1 (Supplementary Figure 1).

#### Protein-protein interaction analysis of targets against Alzheimer’s disease

To explore the relationship between these 131 drug targets, the PPI network was visualized by Cytoscape 3.9.0 software. A total of 131 nodes and 1,563 edges and an average node degree of 24 were obtained. The darker the color, the larger the node, and the greater the degree value (Supplementary Figure 2). Using the maximum cluster centrality (MCC) algorithm of the cytoHubba plug-in, the top 70 hub genes with the highest degree were calculated according to degree centrality, betweenness centrality, and closeness centrality. The MCC algorithm captures more essential proteins in the top-ranked list in both high- and low-degree proteins, with better performance for key protein predictions (25). Therefore, the top 70 hub genes under this algorithm were considered to be more conducive to the subsequent biological enrichment analysis.

#### Gene ontology and kyoto encyclopedia of genes and genomes enrichment analysis

The top 70 hub genes were further analyzed by Metascape to facilitate functional prediction. Through GO analysis, screening was carried out under the condition of log *P* ≤ −10 (Supplementary Figure 3). In the biological process (BP), the positive regulation of protein phosphorylation, response to oxidative stress, cellular response to chemical stress, and positive regulation of kinase activity were mainly involved. The molecular functions (MF) of these GO terms included protein kinase activity, phosphotransferase activity, the alcohol group as an acceptor, transmembrane receptor protein kinase activity kinase binding, and protein kinase binding. Membrane raft, focal adhesion, receptor complex, vesicle lumen, and cell-substrate junction were involved in cellular component (CC).

Additionally, KEGG pathway enrichment analysis demonstrated that these 70 hub genes contributed to 245 pathways (*P*-value < 0.05). Based on relevant literature reports, the top 20 signaling pathways closely related to AD were screened (Supplementary Figure 4). It mainly involves the PI3K-Akt signaling pathway, EGFR tyrosine kinase inhibitor resistance, MAPK signaling pathway, AGE-RAGE signaling pathway in diabetic complications, fluid shear stress and atherosclerosis, estrogen signaling pathway, Ras signaling pathway, and prolactin signaling pathway.

#### Component-target-pathway network analysis and key target screening

**FIGURE 6:**
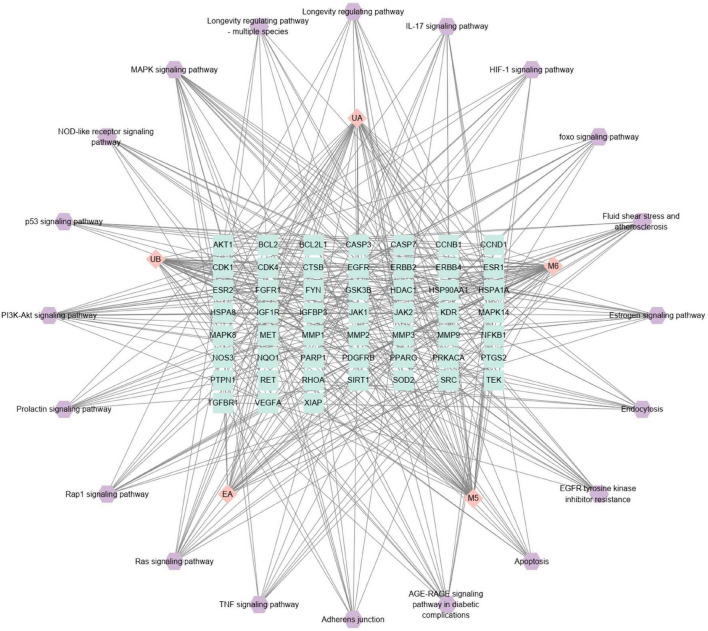
Construction of component-target-pathway (C-T-P) network. The C-T-P network of the top 20. The pink diamond represented the compounds, and the green square was the target proteins associated with AD-related pathway (purple hexagon).

To reveal the interactions between components and targets and related pathways for the treatment of AD, a C-T-P network based on the component-target-pathway was constructed. As illustrated in Figure 6, the C-T-P network consisted of 77 nodes (5 compounds and 52 targets on 20 AD-associated pathways) and 393 edge interactions. Through the analysis of the C-T-P network, AKT1, IGF1R, NFKB1, EGFR, and MAPK14 were identified with high degree value as the key targets of the network (Table 2), indicating that these targets were closely related to other targets and pathways, i.e., they are the key factors of EA and urolithins against AD. These targets collectively participate in the PI3K-Akt signaling pathway, MAPK signaling pathway, Ras signaling pathway, fluid shear stress and atherosclerosis, and EGFR tyrosine kinase inhibitor resistance, suggesting that these targets may play important roles in the treatment of AD and jointly interfere with disease progression.

**TABLE 2:** The key AD-associated targets of ellagic acid and urolithins.

Gene name	Protein name
AKT1	RAC-alpha serine/threonine-protein kinase
IGF1R	Insulin-like growth factor 1 receptor
NFKB1	Nuclear factor NF-kappa-B p105 subunit
EGFR	Epidermal growth factor receptor
MAPK14	Mitogen-activated protein kinase 14

From the results of the C-T-P network, the PI3K-Akt signaling pathway and MAPK signaling pathway are regarded as the most prominent pathways through which EA and urolithins to affect AD, and these pathways are associated with 21 and 19 key targets, respectively. High expression of PI3K and Akt has been reported to induce Bad phosphorylation, which is involved in suppressing oxidative stress-induced cell death and promoting cell survival associated with neurodegenerative diseases such as AD and PD (26). Therefore, activation of PI3K-Akt is considered to be a beneficial strategy for the treatment of AD. A report by Chen et al. revealed that UB ameliorates cognitive deficits by promoting neuronal survival in aging mice through the PI3K pathway. This effect is achieved through down-regulation of the c-Jun N-terminal kinase (JNK) signaling pathway and activation of ERK and phosphoinositide 3-kinase (PI3K), which inhibits neuronal apoptosis through phosphorylation and activation of Akt and P44/42 mitogen-activated protein kinase (MAPK) (27). These results indicate that the MAPK signaling pathway may play a crucial role in the intervention of EA and urolithins in AD. Similarly, Xu et al. found that UA and UB exerted anti-inflammatory activity in LPS-induced BV2 microglia by inhibiting the NF-κB, MAPK and PI3K-AKT signaling pathways. It was determined that the hyperphosphorylation levels of Erk1/2, p38 MAPK and Akt induced by LPS in BV2 microglia were significantly reduced after treatment with UA and UB (28, 29).

These clues suggest that UA, UB, UM5, and UM6 may be the real bioactive metabolites of EA, share a common mechanism of action, and exhibit neuroprotective biological activities. The finding suggests the existence of the multiple synergistic effects of EA and urolithins on AD via multiple targets and numerous signaling pathways. Nonetheless, the specific mechanisms and molecular targets remain to be further verified. However, through network pharmacology, we can systematically summarize and predict the relevant pathways that are likely to be regulated by compounds, provide guidance and suggestions for follow-up research work, and accelerate target discovery and mechanism elucidation.

### Validation of molecular docking

**FIGURE 7:**
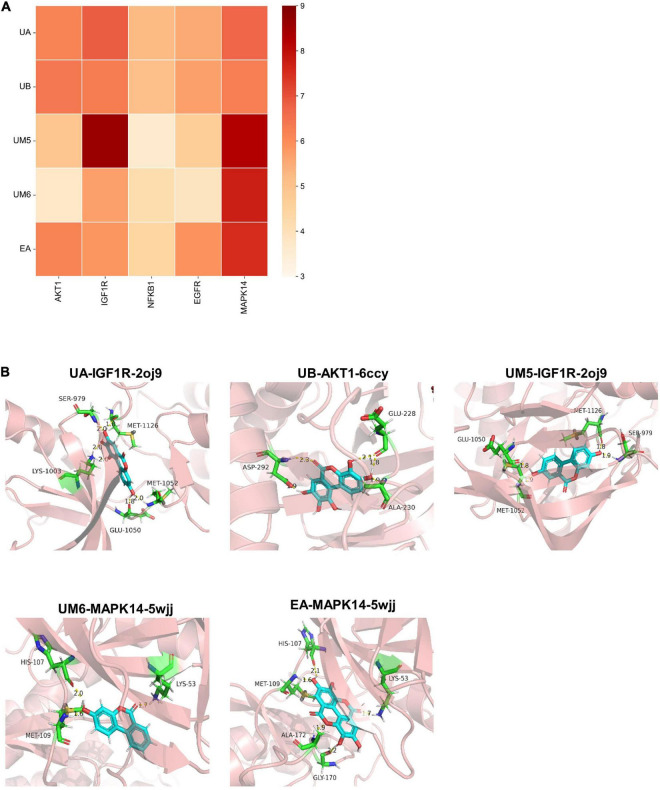
Molecular docking and visualization of docking results. **(A)** Heat map of docking results. **(B)** Visualization of binding patterns of EA and urolithins to key targets.

Furthermore, according to the predicted key target information on EA and urolithins in AD intervention, molecular docking and docking mode visualization were carried out, respectively. Based on the predictive results of network pharmacology, a total of 5 target genes, namely AKT1, IGF1R, NFKB1, EGFR, and MAPK14, which showed strong interactions with other targets, pathways and components, were screened out (Table 2). As shown in the docking scores (Table 1) and their representative heatmap of docking results (Figure 7A), all targets exhibited good binding affinity with compounds (score ≥ 5). Subsequently, the action modes of the compounds targeted by the five proteins were visualized (Figure 7B). We found that UA and UM5 mainly interact strongly with IGF1R, while UB showed good binding activity with AKT1. Meanwhile, both UM6 and EA have high binding affinity with MAPK14. Notably, UM5 exhibited strong interactions with the targets IGF1R and MAPK14. UM5 forms a stable complex with IGF1R through four hydrogen bonds with amino acid residues Glu-1050, Ser-979, Met-1052, and Met-1126. The results showed that different molecules can produce strong binding affinity with the targets, and the simulation results verify the binding activity of small molecules with the target. Computer simulation verification provides a reference for target verification and pathway focusing.

The bioavailability of urolithins has been fully confirmed, and they exhibited good biosafety and bioactivity in many studies, including neuroprotective effects in neurodegenerative diseases (30–32). UA has been reported to inhibit mitochondrial dysregulation and prolong lifespan in *C. elegans* (18). Moreover, Chen et al. found that UB could ameliorate cognitive deficits by inhibiting Cyt C-mediated apoptosis and promoting neuronal survival in aging mice through the PI3K pathway (27). Thus, the neuroprotection-related biological activities of EA, UA, and UB have been widely investigated, whereas the activity comparison and effect relationship between EA and other urolithin monomers remain to be elucidated. Based on this, we supplemented the research on the neuroprotective activity of urolithin UM5 and UM6.

In summary, the anti-Aβ_25–35_-induced neurotoxicity of EA and urolithins indicates the potential therapeutic drug candidates for AD treatment. Nevertheless, it is necessary to carry out further *in vivo* studies and/or *in vitro* validation experiments in follow-up work. The anti-Aβ_25–35_ effect of EA and urolithins suggests that they are potential therapeutic candidates for the treatment of AD. Nevertheless, it is necessary to further investigate the positive effects of EA and urolithins on AD through *in vivo* and/or *in vitro* experiments. Additionally, it is worth noting that due to the differences in intestinal microorganisms, some populations cannot normally produce UA or UB terminal metabolites and, can only metabolize to UM5. Therefore, although UA and UB exhibit better blood brain barrier (BBB) permeability, the upstream metabolites of UM5 and UM6 due to individual differences should not be ignored. Existing studies have shown that EA may produce neuroprotective-related biological effects in humans through its urolithin metabolites (9, 32). This study further indicates that UM5 and UM6 possess the potential of anti-AD natural inhibitors similar to EA, UA, and UB, providing new insights for elucidating the pharmacological activities of plant polyphenols EA and urolithin metabolites. In addition, our results demonstrate at the cellular level that even a subset of people who cannot naturally metabolize UA or UB end-metabolites due to differences in gut microbiota may still exert neuroprotective health benefits through UM5 and UM6 after ingesting of dietary polyphenol EA (33, 34). This notion is confirmed by our study results, filling a gap in the research on the neuroprotective activity of UM5 and UM6. Overall, UM5 and UM6 have similar biological activities to those of UA and UB and can also be used as potential intervention drugs for AD, but more in-depth research data are needed to supplement and support these findings.

## Conclusion

The importance of an early intervention for AD has been emphasized along with the advances in diagnostic technologies. Due to their safety and multiple effects, natural products and their bioactive metabolites, therefore, play important roles in early stage AD therapy. The current study revealed that a widely distributed natural compound EA and its *in vivo* metabolites could significantly improve the mitochondrial energy metabolism, and attenuate the Aβ-induced toxicity in PC12 cells. This neural protective effect also accelerated the outgrowth of neurites in a primary neuron model. These results provide evidence of the potential anti-AD effect of EA and urolithins, especially for UM5 and UM6. The possible action mechanisms of those compounds involved in the AD therapy were predicated to be correlated with PI3K-Akt, MAPK, and Ras signaling pathways. In this study, we have pioneered and supplemented studies on the neuroprotective activity of UM5 and UM6, and demonstrated that they can be considered as potential drugs for AD, but further in-depth validation is still required.

## Data availability statement

The original contributions presented in the study are included in the article/Supplementary material, further inquiries can be directed to the corresponding author/s.

## Author contributions

H-LL: investigation, manuscript preparation, and diagramming. S-YZ: bioactivity evaluation and revise. X-HP: methodology and investigation. Y-SR: bioactivity evaluation. J-CZ and Y-XZ: visualization and software. W-ZH: resources. Z-YY: methodology and review and editing. S-MW: supervision and platform support. Y-WG: supervision, project administration, and review and editing. All authors read and approved the final manuscript.

## Footnotes

1
https://pubchem.ncbi.nlm.nih.gov

2
http://www.swisstargetprediction.ch

3
http://lilab-ecust.cn/pharmmapper

4
https://www.genecards.org

5
https://mirror.omim.org

6
http://metascape.org

7
http://www.rcsb.org/pdb/
