# Supplementary material for: Identification of ellagic acid and urolithins as natural inhibitors of Aβ25–35-induced neurotoxicity and the mechanism predication using network pharmacology analysis and molecular docking
Source: Front Nutr. 2022 Aug 2;9:966276. doi: 10.3389/fnut.2022.966276 (PMC9378864; doi:10.3389/fnut.2022.966276)
Supplement: Supplementary file 1 [file Data_Sheet_1.PDF]

Supplementary Material

Identification of Ellagic Acid and Urolithins as Natural Inhibitors of Aβ25-35-Induced Neurotoxicity and the Mechanism Predication Using Network Pharmacology Analysis and Molecular Docking

Hui-Lin Li[1,2,3,4, †], Shi-Ying Zhang[1,2,3, †], Ying-Shan Ren[1,2,3], Jie-Chun Zhou[1,2,3], Ying-Xin Zhou[1,2,3], Wei-Zhong Huang[5], Xiu-Hong Piao[1,2,3,6], Zhi-You Yang[7,*], Shu-Mei Wang [1,2,3,*], Yue-Wei Ge[1,2,3,*]

[1] *School of Chinese Materia Medica, Guangdong Pharmaceutical University, Guangzhou 510006, China*
[2] *Key Laboratory of Digital Quality Evaluation of Chinese Materia Medica of State Administration of TCM, Guangdong Pharmaceutical University, Guangzhou 510006, China*
[3] *Engineering & Technology Research Center for Chinese Materia Medica Quality of the Universities of Guangdong Province, Guangdong Pharmaceutical University, Guangzhou 510006, China*
[4] *Macau University of Science and Technology, Macau 999078, China*
[5] *Guangdong Luofushan Sinopharm Co., Ltd, Huizhou 516133, China*
[6] *School of Life Sciences and Biopharmaceutics, Guangdong Pharmaceutical University, Guangzhou 510006, China*
[7] *Guangdong Provincial Key Laboratory of Aquatic Product Processing and Safety, College of Food Science and Technology, Guangdong Ocean University, Zhanjiang 524088, China*
*Correspondence:
Tel.: +86-20-39352177.
E-mail: geyuewei@gdpu.edu.cn (Yue-Wei Ge); gdpuwsm@126.com (Shu-Mei Wang); yang_zhiyou@sina.com (Zhi-You Yang)
Addresses: Waihuan East Road 280, Guangzhou Higher Education Mega Center, Guangzhou 510006, Guangdong, China
[†], These authors contributed equally to this paper and share co-first authorship.

Content

Figure S1. Venny diagram of disease and drug targets.

Figure S2. The protein-protein interaction (PPI) network of common targets.

Figure S3. Gene Ontology (GO) analysis of biological process (BP), cellular component (CC) and molecular function (MF).

Figure S4. Analysis of Kyoto Encyclopedia of Genes and Genomes (KEGG) pathway enrichment.

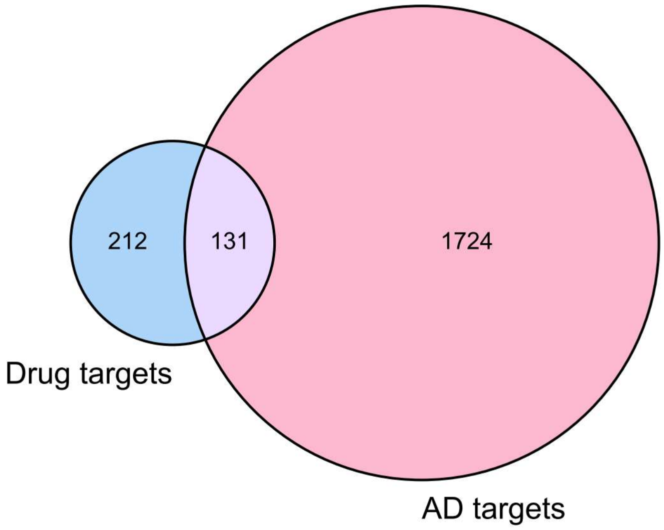

Figure S1. Venny diagram of disease and drug targets.

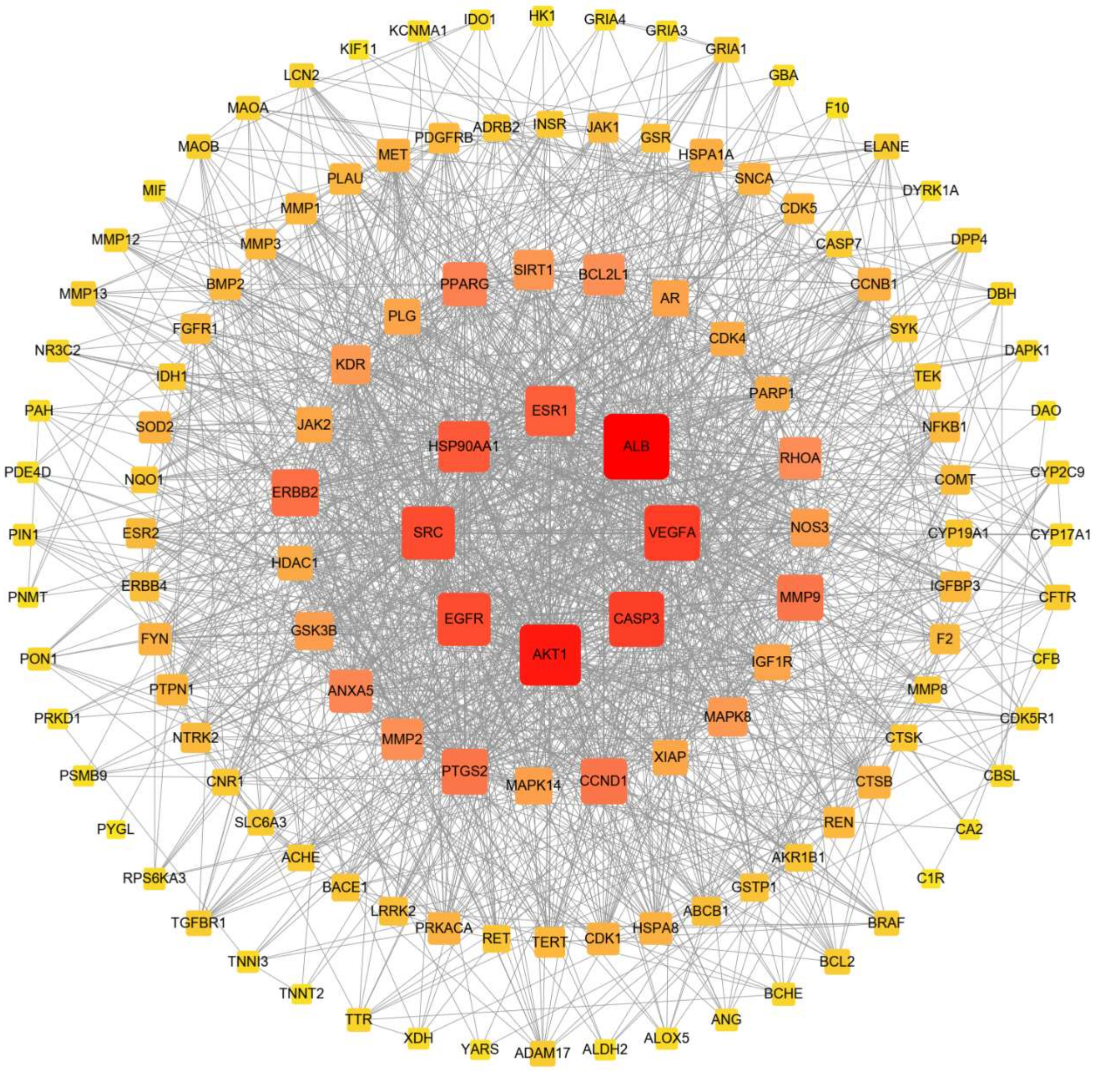

Figure S2. The protein-protein interaction (PPI) network of common targets.

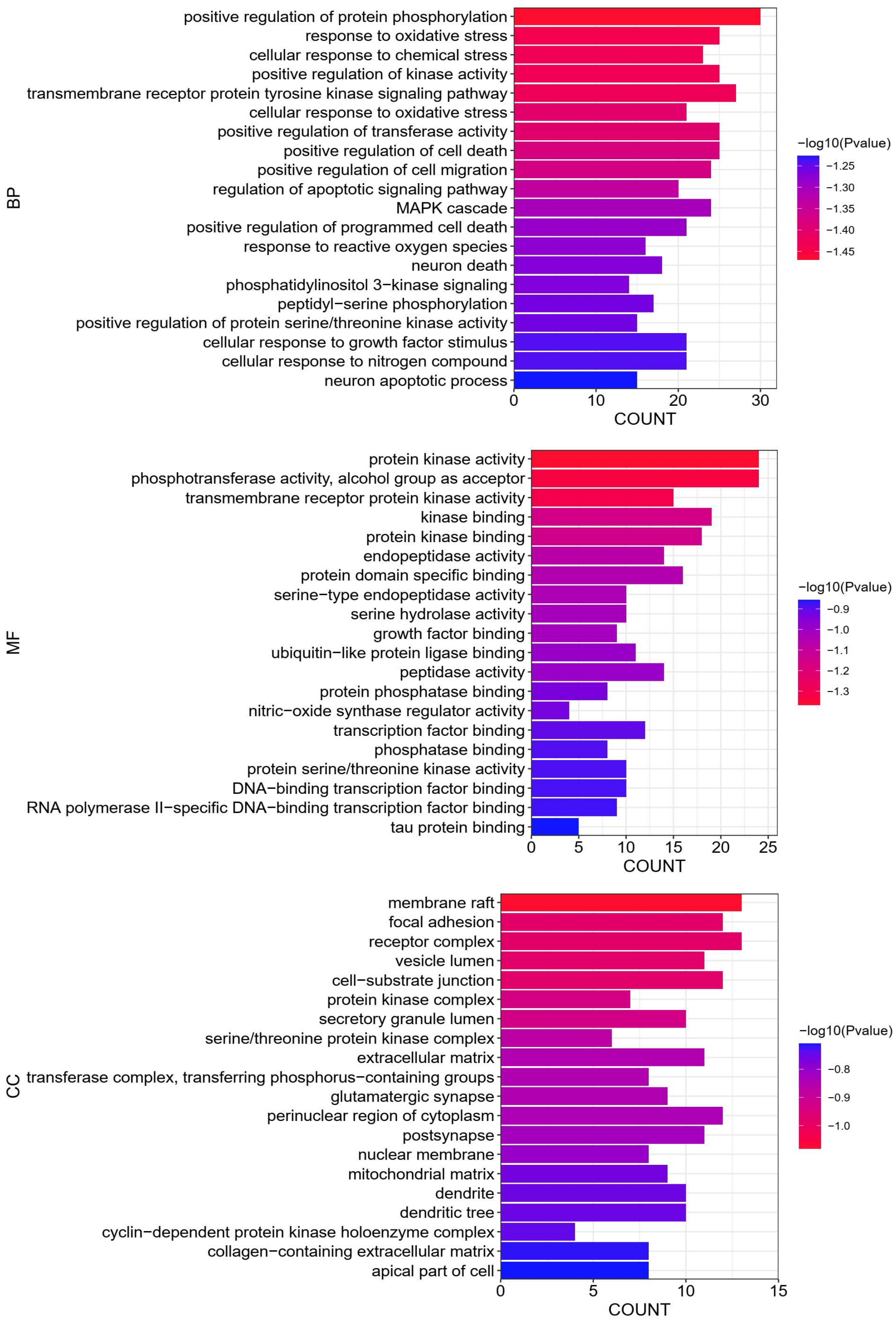

Figure S3. Gene Ontology (GO) analysis of biological process (BP), cellular component (CC) and molecular function (MF).

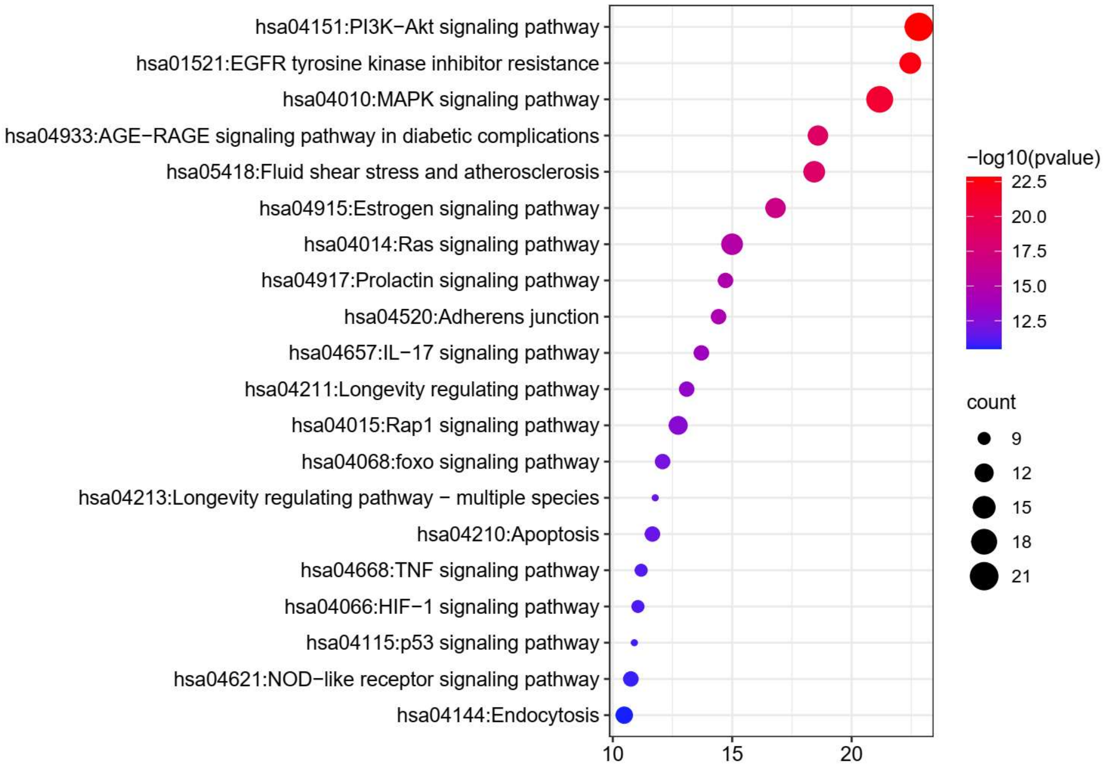

Figure S4. Analysis of Kyoto Encyclopedia of Genes and Genomes (KEGG) pathway enrichment.
